# Current advances in seagrass research: A review from Viet Nam

**DOI:** 10.3389/fpls.2022.991865

**Published:** 2022-10-10

**Authors:** Xuan-Vy Nguyen, Thi Thuy Hang Phan, Van-Luong Cao, Nhu-Thuy Nguyen Nhat, Trung-Hieu Nguyen, Xuan-Thuy Nguyen, Va-Khin Lau, Cong-Tin Hoang, My-Ngan Nguyen-Thi, Hung Manh Nguyen, Viet-Ha Dao, Mirta Teichberg, Jutta Papenbrock

**Affiliations:** ^1^ Institute of Oceanography, Viet Nam Academy of Science and Technology, Nha Trang, Vietnam; ^2^ Faculty of Marine Science and Technology, Graduate University of Science and Technology, Ha Noi, Vietnam; ^3^ University of Sciences, Hue University, Hue, Vietnam; ^4^ Institute of Marine Environment and Resources, Viet Nam Academy of Science and Technology, Hai Phong, Vietnam; ^5^ Dead Sea and Arava Science Center, Central Arava Branch, Hatseva, Israel; ^6^ French Associates Institute for Agriculture and Biotechnology of Dryland, The Jacob Blaustein Institutes for Desert Research, Ben-Gurion University of the Negev, Sede Boqer Campus, Midreshet Ben-Gurion, Israel; ^7^ Ecosystems Center, Marine Biological Laboratory (MBL), Woodshole, MA, United States; ^8^ Institute of Botany, Leibniz University Hannover, Hannover, Germany

**Keywords:** degradation, distribution, diversity, ecosystem services, seagrass, taxonomy

## Abstract

Seagrass meadows provide valuable ecosystem services but are fragile and threatened ecosystems all over the world. This review highlights the current advances in seagrass research from Viet Nam. One goal is to support decision makers in developing science-based conservation strategies. In recent years, several techniques were applied to estimate the size of seagrass meadows. Independent from the method used, there is an alarming decline in the seagrass area in almost all parts of Viet Nam. Since 1990, a decline of 46.5% or 13,549 ha was found. Only in a few protected and difficult-to-reach areas was an increase observed. Conditions at those sites could be investigated in more detail to make suggestions for conservation and recovery of seagrass meadows. Due to their lifestyle and morphology, seagrasses take up compounds from their environment easily. Phytoremediation processes of *Thalassia hemprichii* and *Enhalus acoroides* are described exemplarily. High accumulation of heavy metals dependent on their concentration in the environment in different organs can be observed. On the one hand, seagrasses play a role in phytoremediation processes in polluted areas; on the other hand, they might suffer at high concentrations, and pollution will contribute to their overall decline. Compared with the neighboring countries, the total C*_org_* stock from seagrass beds in Viet Nam was much lower than in the Philippines and Indonesia but higher than that of Malaysia and Myanmar. Due to an exceptionally long latitudinal coastline of 3,260 km covering cool to warm water environments, the seagrass species composition in Viet Nam shows a high diversity and a high plasticity within species boundaries. This leads to challenges in taxonomic issues, especially with the *Halophila* genus, which can be better deduced from genetic diversity/population structures of members of Hydrocharitaceae. Finally, the current seagrass conservation and management efforts in Viet Nam are presented and discussed. Only decisions based on the interdisciplinary cooperation of scientists from all disciplines mentioned will finally lead to conserve this valuable ecosystem for mankind and biodiversity.

## Introduction

Seagrasses are marine angiosperms that have recolonized the marine habitat approximately 100 million years ago during at least three events (Les et al., 1997). Seagrasses are found in thousands of kilometers of the sedimentary shorelines ranging from tropical to temperate regions. They are found in different aquatic conditions including hypersaline, marine or brackish water at estuarine, nearshore, and subtidal and intertidal sand (Short et al., 2007). They are foundation species and provide essential ecosystem services, e.g., oxygen production, habitat providers, nutrient recycling, among many others (Orth et al., 2006; Fourqurean et al., 2012; Lamb et al., 2017), and represent one of the most significant natural carbon sinks on Earth (Fourqurean et al., 2012; Macreadie and Hardy, 2018). Nonetheless, the seagrass population is suffering a global decline, driven mainly by the growing number of pressures linked directly to human activities (e.g., ocean warming, coastal modification, water quality degradation) (Orth et al., 2006; Waycott et al., 2009). Globally, seagrasses are disappearing at a worrying rate of 110 km^2^ per year (Waycott et al., 2009). Based on review from 215 different studies, Waycott et al. (2009) revealed that 29% of the total world seagrass population was lost from 1980 to 2006. Moreover, seagrass ecosystems in Japan, Europe, Australia, and USA have been lost as a result of diseases, deteriorated water quality, and coastal development (Sullivan et al., 2018).

In the Southeast Asia (SEA) region, the review study of Fortes et al. (2018) estimated that the total seagrass cover was about 36,763 km^2^. However, McKenzie et al. (2020) indicated that the global seagrass distribution is much lower than that mentioned in previous publications. Sudo et al. (2021) showed that the seagrass distribution in the SEA was about 3,670 km^2^. Seagrass bed decline was found in almost all countries from SEA. Recently, Sudo et al. (2021) combined data from Global Distribution of Seagrasses (GDS) issued by UNEP-WCMC (before 2001), and new data (Sudo and Nakaoka, 2020) from 68 sites in nine countries/regions of SEA showed that more than 60% of seagrass meadows declined at an average rate of 10.9% year^−1^, while 20% of beds increased at an average rate of 8.1% year^−1^. Therefore, an overall average decline of 4.7% year^−1^ in SEA has been estimated. In particularly, in reports on the status of seagrass beds from Indonesia, Unsworth et al. (2018) indicated that seagrasses across the Indonesian archipelago are in a critical state of decline. In Malaysia, loss of seagrass habitats was recorded at different specific sites (Hossain et al., 2015; Bujang et al., 2016). However, a recent study on a smaller scale at Nakhon Si Thammarat Province, Thailand, showed an increasing area of seagrass beds (Rattanachot et al., 2018). Therefore, there is an urgent need to map existing intertidal seagrasses in Thailand and elsewhere to better understand reasons for both decrease and increase in seagrass meadows (Koedsin et al., 2016). Among the SEA region, Fortes et al. (2018) reported that there are 21 seagrass species, but some of these are still considered taxonomically uncertain. Seagrass species richness from SEA is the highest in the world (Short et al., 2011). Since the last decade, the increase in use of genetic markers has successfully solved some issues in taxonomy and genetic diversity, population structure, and gene flows among seas/oceanic systems (Nguyen et al., 2014; Arriesgado et al., 2015; Wainwright et al., 2018).

Based on the status review, we focus on current research and highlight gaps in knowledge of seagrass ecosystems within Viet Nam. First, the current seagrass distribution from Viet Nam and changes in selected sites are described, including the important role in phytoremediation processes by seagrass. The role of seagrass meadows for blue carbon storage are also discussed with an emphasis on the situation in Viet Nam. Next, the taxonomic issues of *Halophila* and genetic diversity of members of Hydrocharitaceae collected from the Vietnamese waters are presented. Finally, the importance of the interdisciplinary cooperation of scientists from all disciplines is discussed for future works.

## Seagrass distributions and changes

**Figure 1:**
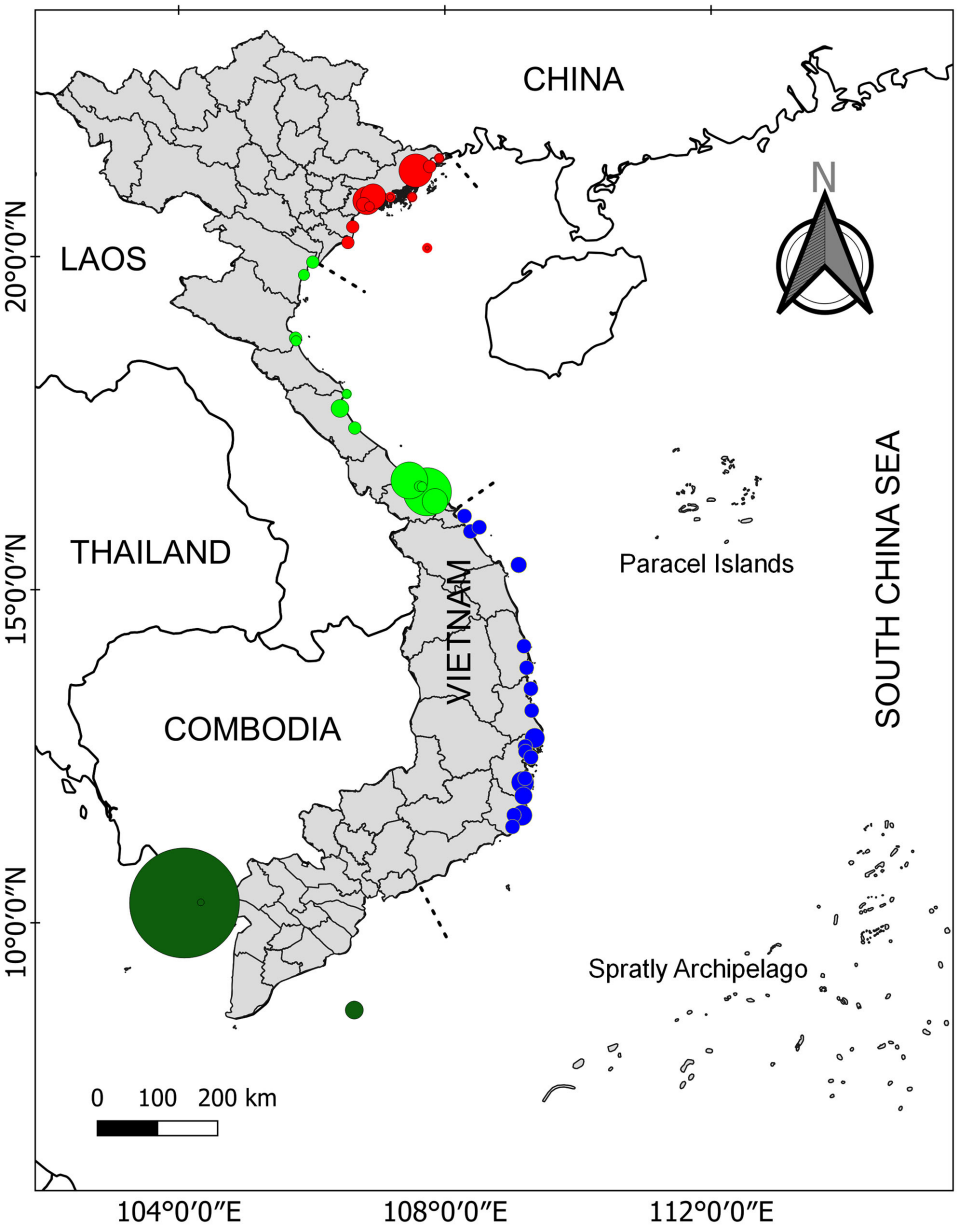
Seagrass distribution in Viet Nam. The four different color cycles indicate seagrass distribution in four different regions. Data were combined from various studies. See **Table 1** for more details.

Viet Nam is located in the central part of SEA where it is known as the place of the evolutionary origin of seagrasses (Chen et al., 2012). Among 22 ecoregions of seagrass distribution from SEA, Viet Nam consists of three ecoregions including N_0_20112 (Gulf of Tonkin), N_0_20115 (Gulf of Thailand), and N_0_20116 (southern Viet Nam) (Spalding et al., 2007). The coastline of Viet Nam was divided into four regions including northeast (1), north central (2), south central (3), and southern Viet Nam (4) (see **Figure 1** for more details). Cao et al. (2014) reported that in the total area of 2,240 ha, 12 seagrass meadows were found in region 1. For region 2, two seagrass meadows were localized at Tam Giang-Cau Hai lagoon and a nearby area with 2,037 and 618 ha, respectively (Cao et al., 2014; Cao et al., 2020a). For region 3, numerous geographically suitable areas (e.g., lagoons, bays, islands, islets, atolls, and reefs) can be identified along the coast providing a diversity of habitats for the occurrence of seagrasses. The seagrass distribution of region 3 is the most well-studied among the four regions with a total area of 3,109 ha (Cao, 2011; Cao et al., 2019; Nguyen et al., 2021a; Nguyen et al., 2021b). Recently, Nguyen et al. (2021a) showed that the seagrass distribution at Phu Quoc Island (i.e., the biggest island of Viet Nam) was about 7,579 ha and is considered the biggest seagrass bed in Viet Nam (region 4). In addition, another recent study has documented the existence of a 30-ha seagrass bed at Hai Tac archipelago (Do et al., 2020). In **Table 1**, more details on the distribution of seagrass meadows in Viet Nam are illustrated.

**Table 1:** Seagrass distribution (ha) and species diversity at each region.

Regions	Number of provinces/cities	Distribution (ha)	Species diversity	Sources
1	5	2,240	Hb, Ho, Rm	(Cao et al., 2014)
2	6	2,655	Hb, Hu, Rm, Zj	(Cao et al., 2014; Cao et al. (2020a)
3	8	3,109	Hb, Hd, Ho, Hma Hmi, Hsp, Ea, Th, Tc, Cr, Cs, Hu, Hp, Si, Rm	(Nguyen et al., 2021b), (Cao et al., 2019; Cao et al., 2020b; Nguyen et al., 2021a)
4	9	7,609	Hb, Hd, Ho, Hma “Hmi”, Hsp, Ea, Th, Cr, Cs, Hu, Hp, Si	(Do et al., 2020), (Nguyen et al., 2021b), (Nguyen et al., 2021a)
**Total**	**28**	**15,613**		

Hb, *Halophila beccarii*; Hd, H. *decipiens*; Ho, *H. ovalis*; Hma, *H. major; Hmi*, *putative H. minor*; Hsp, *Halophila major SL type*; Ea, *Enhalus acoroides*; Th, *Thalassia hemprichii*; Tc, *Thalassodendron ciliatum*; Cr, *Cymodocea rotundata*; Cs, *C. serrulata*; *Hu, Halodule uninervis*; Hp, H. pinifolia; Si, *Syringodium isoetifolium*; Rm, *Ruppia maritima*. Zj, *Zostera japonica*. See **Figure 1** for the location of the regions.

**Figure 2:**
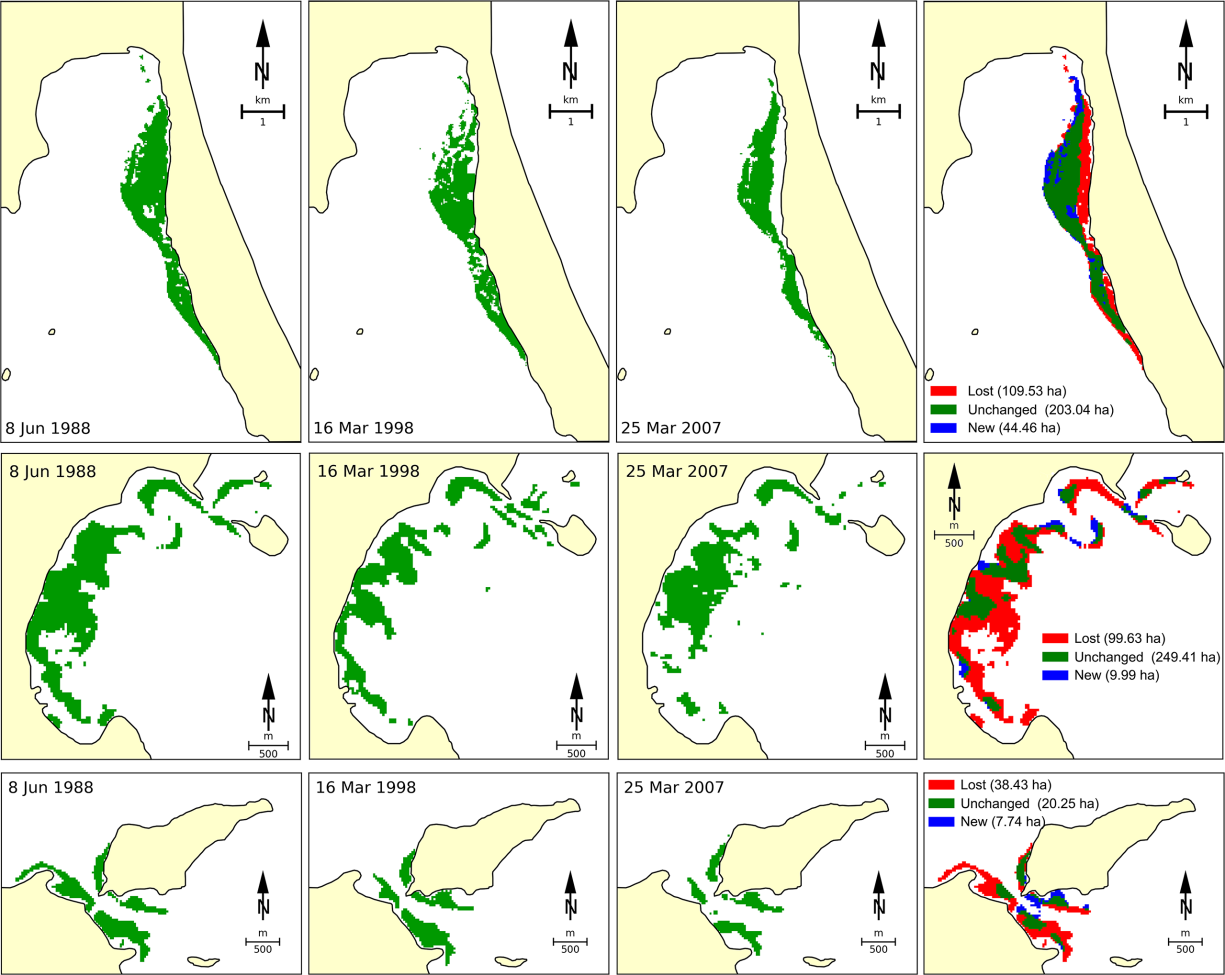
Changes of seagrass beds at three sites of Van Phong Bay (region 3) from 1998 to 2018 deduced by using datasets of satellite image Landsat 5 TM and Landsat 8 OLI. The figure was adapted from Vo et al. (2020).

A significant decline in seagrass coverage from Vietnamese waters has been detected across the whole area. The total seagrass area in Viet Nam was estimated to cover about 29,162 ha in 1990 (Trinh and Takeuchi, 2019). By using satellite Landsat TM/OLI image analysis, Vo et al. (2020) indicated that 186.2 ha (equivalent to 35.8%) of the original seagrass beds were lost in the last three decades at Van Phong Bay due to a number of different reasons (**Figure 2**). The authors identified that typhoons may be the main driver for the loss of seagrass beds at open-sea sites, while human-induced stressors, such as aquaculture activities, excavation, and terrigenous obliteration, may be the main reasons in protected sites. By using Landsat TM/ETM +/OLI imageries and the ground reference data, Chen et al. (2016) indicated that from 1996 to 2015, the total area of seagrass beds in Cam Ranh Bay had declined by approximately 25% (66 ha), mainly due to coastal development and infrastructure construction. Based on Sentinel-2, Landsat-8, and VNREDSat-1 analyses for the Khanh Hoa coastal area (a part of region 3), submerged aquatic vegetation including seagrass was reduced by 74.2%, while gains in new areas compensated for less than half of these losses (Khanh Ni et al., 2020).

Establishing accurate seagrass distribution maps and long-term monitoring, therefore, are needed to form the basis for the conservation and development of the current and future seagrass ecosystems in Viet Nam (Nguyen et al., 2021a; Nguyen et al., 2022a). Furthermore, recent studies of seagrass distribution from Viet Nam may partly fulfill knowledge gaps on basic information on seagrass habitats in southeast Asia, as mentioned by Fortes et al. (2018). New methods such as spectroscopic methods with a higher resolution of remote sensing images will be helpful to determine species composition within seagrass beds. Although Nguyen et al. (2021) mapped almost all seagrass beds of a significant size from South Viet Nam, the patchy small-scale meadows from offshore islands, lagoons, and inlets were not included. In addition, *Halophila decipiens* beds in deep waters such as close to the Cu Lao Cham Islands, offshore Ninh Thuan (>15 m) may not be detected in remote sensing imageries. Therefore, a combination of several approaches including ultra-high-resolution, multibeam echo-sounder, unmanned aerial vehicles (UAVs) and fieldwork are needed to map seagrass beds in deep waters.

## Biodiversity in seagrass beds

Studies of biodiversity of fishes within seagrass beds revealed differences in different habitats and locations. In region 1, results of Nguyen (2013) indicated that the density of fish larvae and juveniles within seagrass beds (327 individuals m^−2^) was 180 times higher than the bare sediment at Cat Ba Island (1.79 individuals m^−2^). Taxon diversity of fishes within seagrass beds in this site was only six taxa. For the crustaceans, there is a number of taxa of juveniles including groups of Penaiedae, Alpheidae, Palaemonidae, Aryidae, Squillidae, Sergestidae, and Pandalidae that were commonly found. The density and biomass of zoobenthos showed high variation between seagrass beds and the bare sediment in both dried and rainy seasons. In the rainy season, the density and biomass of zoobenthos in seagrass beds were 434 (individuals m^−2^) and 169.9 (g m^−2^), respectively, whereas those values were 128 and 27.6 in the bare sediment. In the dry season, the density and biomass of zoobenthos in seagrass beds were much higher with 1,226 (individuals m^−2^) and 289.3 (g m^−2^) (Nguyen et al., 2002).

In region 2, seagrass beds in Tam Giang-Cau Hai lagoon are the largest size. Therefore, there are several studies on animals’ diversity including fishes, crustacean, mollusk, zoo, and phytoplankton. Eighty-seven taxon of fish larvae and juveniles were identified from this lagoon. Among them, some high economic species such as *Epinephelus sexfasciatus* Valenciennes, *Lujianus russelii* Bleeker, *Lethrinus* spp., and *Siganus* sp. are the most dominant. Nguyen (2013) showed that there is a positive correlation between above-ground biomass of seagrass and diversity of fish larvae and juveniles in almost all seagrass beds occurring in Tam Giang-Cau Hai lagoon. In addition, Nguyen and Nguyen (2012) listed 177 fish species at Tam Giang-Cau Hai lagoon, the highest species composition compared to other locations. Based on underwater videos, Espadero et al. (2020) identified 59 fish species representing 23 families that were recorded in the 26 video deployments in the seagrass beds from the Philippines. For Crustaceans, 20 taxa of crustacean larvae and juveniles were recorded in this lagoon. Among families, both Penaeidae and Portunidae were the dominant groups. For the diversity of zoobenthos, 203 species including 92 species of mollusk, 51 species of crustacean, 49 species of polychaeta, and other species of Echinodermata were found (Nguyen, 2013).

Biodiversity of marine organisms in seagrass beds along the coast of region 3 also showed variation. In general, the diversity of juvenile shrimps and fish species in seagrass beds is higher than in the bare sediment. Nguyen et al. (2000) showed that density of larvae and juveniles of Penaeidae in seagrass beds (78 individuals m^−3^) is eight times higher than the bare sediment (17.12 individuals m^−3^) at Thuy Trieu lagoon. For larvae and juvenile of fish in seagrass beds from the in Cua Dai, the results of Nguyen et al. (2008) indicated that the density of these groups in seagrass beds was 2.8 times higher than that in the bare sediment. Fish species composition within seagrass beds at Thuy Trieu lagoon included 87 species belonging to 12 orders and 47 families. Among them, order Perciformes showed the highest family with 30 families (Nguyen and Nguyen, 2012). In addition, there are 68 species of zoobenthos, with 39 species of polychaeta, 18 species of mollusk, 7 species of crustacean, and 4 species of Echinodermata in seagrass beds in Xuan Tu (Nguyen, 2013). Several studies (Surugiu et al., 2021; Barnes, 2022) revealed that seagrass beds support assemblages of macrobenthic invertebrates with different composition and with considerably greater abundance and species density than adjacent areas without this cover.

Seagrass beds in Phu Quoc Island are considered as typical beds in region 4. There are 33 species of larvae and juvenile fish found within seagrass beds. Higher species composition and diversity of larvae and juvenile fish were found in dense seagrass beds. Twenty species of larvae and juveniles of crustacean were also recorded, and the density of this group was up to 350 individual m^−2^ (Nguyen, 2013). Of the 86 fish species that were found in seagrass beds, the family Apogonidae showed the highest diversity with 18 species. A comparison of diversity of marine organisms including larvae and juveniles and fish composition in three typical seagrass beds (lagoon, estuary, and offshore islands) is presented in **Table 2**. Diversity of zoobenthos in seagrass beds in the southern area (regions 3 and 4, 292 species) was greater than that in the northern area (region 1 and 2, 134 species). Among them, Gastropoda and Bivalvia revealed higher species diversity in the south, whereas species diversity of Annelida and Arthropoda in the North showed higher number of species (**Table 3**).

**Table 2:** Number of families and species of zoobenthos in seagrass beds.

	Tam Giang–Cau Hai^a^	Lap An^a^	Cua Dai^b^	Thuy Trieu^a^	Phu Quy^c^	Phu Quoc^c^
**Larvae and juveniles of fishes**						
Families	47	15	12	n.d.	19	20
Species	87	15	15	n.d.	24	30
**Larvae and juvenile of crustacean**						
Families	6	8	2	n.d.	3	8
Species	20	12	8	n.d.	12	20
**Fishes**						
Families	na	50	32	46	14	34
Species	na	151	55	87	25	86

n.d., not determined. (Source: Nguyen, 2013). ^a^lagoon, ^b^estuary, ^c^offshore island.

**Table 3:** Number of family and species of zoobenthos in seagrass beds.

		Northern part(Regions 1 and 2)	Southern part(Regions 3 and 4)
**Phylum Annelida**			
	Families	18	13
	Species	40	21
**Phylum Mollusca**			
	Class Gastropoda		
	Families	15	24
	Species	31	105
	Class Bivalvia		
	Families	15	24
	Species	38	114
	Class Cephalopoda		
	Families	n.d	1
	Species	n.d	1
**Phylum Echinodermata**			
	Class Asteroidea		
	Families	n.d	4
	Species	n.d	8
	Class Echinoidea		
	Families	n.d	5
	Species	n.d	10
	Class Holothuroidea		
	Families	1	3
	Species	1	12
	Class Ophiuroidea		
	Families	1	1
	Species	2	1
**Phylum Arthropoda**			
	Families	13	10
	Species	22	20
	**Total**	**134 species/63 families**	**292 species/85 families**

n.d., not determined (Source: Nguyen, 2013).

Larvae and juveniles strongly differ in morphology from adults, and their identification to the species level remains problematic. In addition, the current guide of Indo-Pacific fish larvae allows identification only to the family level. DNA barcoding method can identify fish larvae samples to genus and species level (Collet et al., 2018). Identification of fish larvae were best conducted with the aid of molecular method in a study on larval fishes collected from Hawaiian waters (Xing et al., 2022). Therefore, validation of diversity of larvae and juveniles within seagrass beds in Viet Nam may be enhanced when we apply these methods.

## Phytoremediation processes of seagrass

Anthropogenic activities have increased heavy metal pollution in previously uncontaminated ecosystems, threatening terrestrial and aquatic plant communities (Boquete et al., 2021; Zhang et al., 2021). Most heavy metals are not an essential element for plants, and excessive amounts can cause growth inhibition and even death (Burkholder et al., 2007). In Viet Nam, the number of studies on heavy metal accumulation and phytoremediation processes of seagrasses are very limited. A study on accumulation of different heavy metals in the three different organs of the tropical seagrass species *Enhalus acoroides* collected in different lagoons and bays showed that a significant positive correlation of the bio-concentration factor (BCF) for Cu was observed between sediment and rhizome, while significant positive correlations of BCF for Cu, Pb, and Zn were observed between sediment and roots (Nguyen et al., 2017a). Remarkably, the Cu concentration of *E. acoroides* rhizomes collected near shipyards was approximately 140 µg mg^−1^ DW, higher than at other locations at Khanh Hoa province (<20 µg mg^−1^ DW) (Nguyen et al., 2017b). Dung et al. (2014) indicated that Cu showed an extremely severe enrichment in the marine sediments collected near a shipyard. Phytochelatins (PCs) are considered as an important component of the metal detoxifying mechanisms (Ahmad et al., 2019). PCs occur in plants, algae, and some yeast species that grow at high heavy metal concentrations. PCs are translocated within the plant, transported to the vacuole as PC–metal complexes, and stored as high molecular weight PC–metal complexes (Cobbett and Goldsbrough, 2002; Clemens, 2006). PCs, usually with the structure of (1’-Glu-Cys)_n_–Gly (n = 2–11), are glutathione-derived metal-binding peptide. PC_2_ contains two units of gamma-Glu-Cys, while PC_3_ contains three units of gamma-Glu-Cys. The higher the levels of heavy metals that were accumulated in the tissue, the more units of gamma-Glu-Cys were formed (Cobbett and Goldsbrough, 2002). Results showed that higher PC_2_, appearance of PC_3_, and a strong correlation between PC_2_ and Pb concentration were found in the root organ collected from a Pb-contaminated area from southern Viet Nam (Nguyen et al., 2017a) (**Table 4**). Metallothioneins (MTs) are defined as low-M_r_ Cys-rich proteins that bind heavy metals, and nine MT-like sequences from Cu- or Cd-treated *Posidonia oceanica* were isolated and classified into two subgroups (Giordani et al., 2000). It may reveal the roles of MTs in terms of phytoremediation processes of seagrass, which is not well known. Therefore, studies on relationship between heavy metals exposure and expression levels of MTs from some tropical seagrass species are still gaps. Heavy metal accumulation was carried out from *E. acoroides* only, and the heavy metal accumulation of remaining species are still unknown.

**Table 4:** Comparison of concentration of selected heavy metals in the rhizosphere sediment, distinct organs, and phytochelatins (PCs) in *Enhalus acoroides* from different locations along the coast of Khanh Hoa province and other locations.

		Cd	Cu	Pb	Zn	PC_2_	PC_3_
MG-Vie***	Sediment	0.05	40.77	5.08	40.17	n.d	n.d
	Leaf	0.05	10.23	0.08	37.13	0.29	n.d
	Rhizome	0.08	143.90	0.04	28.60	0.24	n.d
	Root	0.07	9.58	1.22	38.12	0.59	n.d
TT-Vie***	Sediment	0.14	6.37	19.90	27.97	n.d	n.d
	Leaf	1.37	2.17	0.08	26.03	0.22	n.d
	Rhizome	0.65	3.86	0.04	14.28	1.13	n.d
	Root	0.59	0.65	19.34	11.64	10.32	0.66
TL-Vie***	Sediment	0.03	1.02	0.81	5.62	n.d	n.d
	Leaf	0.37	2.29	0.08	15.03	0.48	n.d
	Rhizome	0.14	1.47	1.22	13.19	0.80	n.d
	Root	0.16	0.77	4.26	8.87	0.61	n.d
Palau*	Sediment	0.02	8.00	1.00	11.00	n.d	n.d
	Leaf	0.05	3.00	0.50	17.6	n.d	n.d
	Root	0.02	1.10	0.50	8.10	n.d	n.d
India**	Sediment	0.52–5.72	2.76–21.64	4.4–10.36	10.36–127.2	n.d	n.d

MG, My Giang; TT, Thuy Trieu lagoon; TL, Tuan Le; Vie, Viet Nam. Unit = µg g^−1^ DW. n.d., not determined. Source: * Jeong et al. (2021), ** Gopi et al. (2020), *** (Nguyen et al. 2017a).

## Blue carbon storage

Seagrass beds are considered efficient natural carbon sinks among the important coastal blue carbon ecosystems, since they can capture carbon dioxide from the air through photosynthesis and store organic carbon (C*_org_*) in their biomass and within the sediment, thereby mitigating climate change (Fourqurean et al., 2012; Greiner et al., 2013; Macreadie et al., 2019; Williamson et al., 2022). As a result, there is growing interest in managing this “blue carbon” ecosystem worldwide. The C*_org_* content and stocks in seagrass meadows have been thoroughly studied in many countries, and some of them have been developed at a global level to assess “blue carbon,” e.g., the Blue Carbon Initiative. However, basic information on this “blue carbon” habitat is still very limited in Southeast Asia, including Viet Nam (Stankovic et al., 2021).

In recent decades, various methods have been used to measure C fluxes and C storage in seagrass beds, such as measuring techniques of fluxes of oxygen and carbon dioxide, primary production, and remote sensing (Macreadie et al., 2019). However, Viet Nam’s research on carbon storage in seagrasses and other marine plants (e.g., mangroves, seaweeds, and salt marshes) has rarely been published. For example, Cao et al. (2013) calculated the amount of C*_org_* in seagrass beds in Tam Giang-Cau Hai lagoon (central Viet Nam, region 2) through dissolved oxygen (DO) content in a light–dark *ex situ* experiment in the seagrass *Halodule pinifolia*. Specifically, the seagrass ecosystem in the lagoon was estimated to produce 25.71 tons of C day^−1^ during the rainy season and 28.93 tons day^−1^ in the dry season. However, it should be noted that the seagrass beds in the lagoon include up to seven seagrass species, and *H. pinifolia* is not one of the dominant species of the lagoon, so the estimate seems biased.

The carbon storage capacity of seagrasses was also evaluated through their biomass in Thi Nai lagoon (region 3), with the total amount of organic carbon and carbon dioxide fixed by seagrass beds estimated at 136.7 and 501 tons ha^−1^, respectively (Cao and Nguyen, 2017). In addition, Stankovic et al. (2021) recently revealed the potential for seagrass beds’ carbon sinks in Southeast Asia as a nature-based solution for climate change mitigation. The study found that the total C*_org_* (in both sediment and living biomass) in seagrass beds from Viet Nam was 133.16 ± 36.97 Mg ha^−1^. When compared with countries in Southeast Asia, this value was higher than in the Philippines, Malaysia, and Indonesia and almost the same as in Thailand and Myanmar (Stankovic et al., 2021). The local and regional C*_org_* variation among seagrass beds is controlled by many factors such as seagrass community complexity, fine sediment fraction, seawater depth (Monnier et al., 2022; Stevenson et al., 2022), sediment run-off, and primary production of the seagrass (Serrano et al., 2021).

The average total blue carbon stock in seagrass ecosystems in Viet Nam was estimated to be 2.06–2.95 Tg, and these ecosystems can accumulate 25.18–29.28 Gg C*_org_* year^−1^. Compared with the neighboring countries, the total C*_org_* stock from seagrass beds in Viet Nam was much lower than in the Philippines (259.17–425.21 Tg) and Indonesia (62.08–107.50 Tg); however, it was higher than that of Malaysia (0.005–0.25 Tg) and Myanmar (0.02–0.04 Tg) (Stankovic et al., 2021). The difference in average total blue carbon stocks in seagrass ecosystems in different countries depends not only on the total C*_org_* in the seagrass beds but also on the area of seagrass beds. Thus, although Viet Nam has higher C*_org_* in sediments and biomass of seagrass beds than the Philippines and Indonesia, the blue carbon stock from Vietnamese seagrass ecosystems is lower than these two countries because seagrass area of Viet Nam is much less than those two countries (44 and 139 times, respectively) (Stankovic et al., 2021). Thus, studies on blue carbon storage in Vietnamese seagrass beds are very few; further studies such as C*_org_* stock estimates, the carbon accumulation rate, and the risk of loss of C stock in seagrass beds are essential to contribute to comprehensive globe blue carbon estimates for the global climate change mitigation strategy.

## Ecology and physiology of Vietnamese seagrasses related to different environmental conditions

Viet Nam has a long coastline of approximately 3,260 km (exclusive of the shoreline of islands) stretching a latitudinal gradient from Mong Cai (21° 31′ 28.96″ N 107° 57′ 58.28″ E) in the north to Ha Tien (10° 22′ 59.99″ N 104° 28′ 59.99″ E) in the south (Hanh and Furukawa, 2007). Temperature is generally the most important range-limiting factor to seagrass distribution (Duarte et al., 2018). Therefore, the sea water temperature shows variation among regions. In regions 1 and 2, the sea water temperature ranges from 15.7°C to 29°C (average 24.1°C) in the northeast monsoon (November to February) and from 22.6°C to 30.7°C (average 28.5°C) in the southwest monsoon (June to August). However, regions 3 and 4 showed higher sea water temperature in both monsoons; they are 21.6°C–30.7°C (average 27.5°C) and 22.8°C–31.0°C (average 28.9°C) in northeast monsoon and southwest monsoon, respectively (Yu et al., 2019). The difference in sea water between regions 1, 2 and 3, 4 may reflect the species distribution along the coast of Viet Nam. *Zostera japonica* Asch. & Graebn is a common species in both regions 1 and 2 and is found from 13.8°N northward. In contrast, *E. acoroides* (Linnaeus f.) Royle is found from 16°N southward (regions 3 and 4). There are nine species occurring in regions 1 and 2, while more six species are found in regions 3 and 4 (Nguyen, 2013; Nguyen et al., 2021). In addition, the coastal area of Viet Nam consists of numerous bays, estuaries, and beaches with dynamic variabilities in environmental conditions [e.g., depth, sediment characteristics, light, salinity, levels of anthropogenic pressures, seasonal changes, etc. (Tang et al., 2004; Hanh and Furukawa, 2007; Veettil et al., 2020, authors’ observations)]. Therefore, it is expected to have great intra-/inter-species-specific variations in ecological, morphological, and physiological traits among similar/different seagrass species in the country as previously demonstrated in several seagrass species from different regions (Coyer et al., 2004; Jahnke et al., 2019a; Nguyen et al., 2021). To date, however, the number of studies from Viet Nam on this topic remains very limited (Huong et al., 2003; Pham et al., 2006). For example, Huong et al. (2003) investigated seasonal and depth dynamics of two intertidal seagrass species (*Halophila ovalis* and *Z. japonica*) in Ha Long Bay (northern Viet Nam) and demonstrated inter-species specificities between the two species in terms of their tolerances to low-light conditions and desiccation. Interestingly, even occurring in the same area, the two seagrass species sexually reproduced in two distinct timeframes (e.g., November and April for *H. ovalis* and *Z. japonica*, respectively) (Huong et al., 2003). On the other hand, Pham et al. (2006) conducted a year-round monitoring at two nearby bays with different characteristics (especially in depth) in the center of Viet Nam (i.e., Van Phong bay and Cam Ranh bay) focusing on two other seagrass species including *E. acoroides* and *T. hemprichii*. This study showed not only inter-species-specific differences between the two seagrass species from both bays but also intra-specific variations between populations of the same species at both study sites in terms of shoot density, above ground biomass, and leaf production rate (Pham et al., 2006). In a more sheltered environment of a coastal brackish lagoon in central Viet Nam, Phan et al. (2018) revealed that salinity and sediment composition (silk vs. sand) were the two main factors governing the distribution and abundance of seagrasses and other submerged aquatic plants in the lagoon. Overall, studies on the influence of ecological factors on seagrasses in Viet Nam are very few and only small in scope. These pioneer studies have hinted a great dynamic in seagrass ecology of Viet Nam and furthermore emphasize a significant gap in knowledge on this topic to which future studies are strongly encouraged.

## Taxonomic issues of *Halophila*

**Figure 3:**
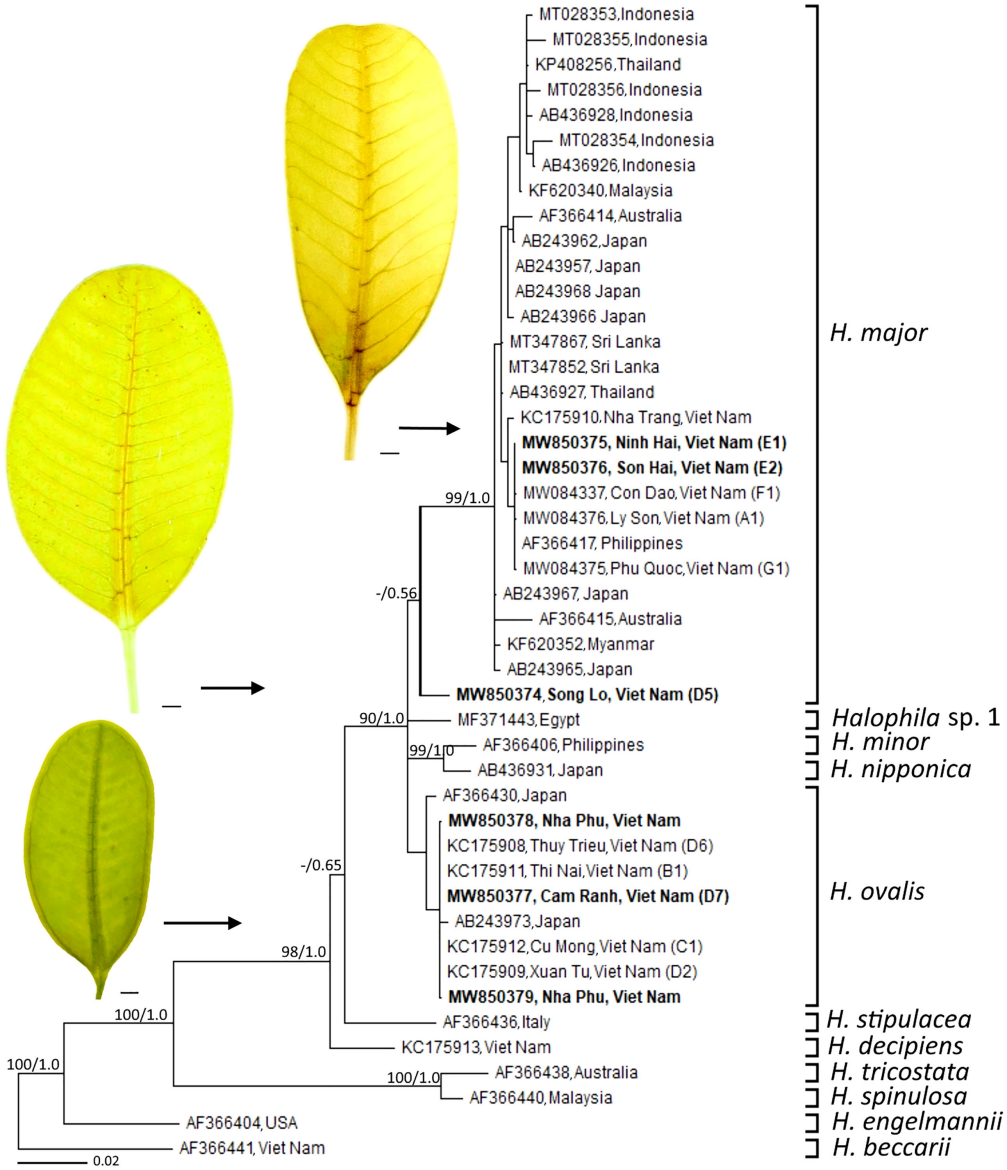
Phylogeny of members of the genus *Halophila* 612 bp of the ITS1-5.8S-ITS2 sequence. Leaf morphology of *Halophila* spp. from samples collected in Viet Nam were placed beside the phylogenetic tree. Scale in each leaf figure = 1 mm. The figure was modified from Nguyen et al. (2021a).

In Viet Nam, the members of *Halophila* were found in different aquatic conditions. *H. beccarii* is found in brackish waters (shallow lagoons), while *H. decipiens* is found in the depth of 5–15 m in offshore islands (Nguyen et al., 2013a; Hoang et al., 2021). The genus *Halophila* is known as one of the most complex taxonomic challenges due to leaf morphological traits that overlap among species (Kuo et al., 2006). *H. johnsonii* Eiseman was first identified as distinct species in paddle-bladed seagrasses of the Hydrocharitaceae (den Hartog, 1970). So far, *Halophila* cf. *johnsonii* was reported from some lagoons in Viet Nam based on the leaf shape (elliptic) (Nguyen et al., 2002). However, the recent detailed analysis of leaf morphology indicated that there were distinctive leaf morphotypes (narrow-leaf type) of *H. ovalis* (Nguyen et al., 2013a). Based on leaf morphology of samples collected from the island of Nha Trang Bay, the trait of distance between intramarginal veins and lamina margin ratio, Nguyen et al. (2013b) introduced the new records of seagrass species—*H. major* for Vietnamese’s flora. *H. minor* was recorded for the first time from Viet Nam by Pham-Hoang (1993). Nguyen et al. (2015) found that there were misidentifications of some samples collected from Vietnamese waters labeled as *H. minor*. It should be treated as *H. ovalis* due to its morphological characteristics and genetic analysis. The misidentification between *H. ovalis* and *H. minor* was also reported from the samples collected at Thailand (Kim et al., 2017). For approximately the last 20 years, no new sequences of *H. minor* have been assigned to GenBank since the study of Waycott et al. (2002). Therefore, the occurrence of *H. minor* is still questionable. Recently, Nguyen et al. (2021a) reported that the main characteristic between *H. ovalis* and *H. major* is that distance between the intra-marginal veins and the lamina margin of *H. ovalis* is much wider than *H. major*, and the intra-marginal veins of *H. ovalis* is easily recognized by naked eyes, and other parameters including leaf width, leaf length, number of cross-veins, number of branching cross-veins, space between cross-veins, and the angle between cross veins and mid-veins did not show significant differences between two species. For different habitat types, *H. ovalis* grows in lagoons that experience large salinity differences between the dry and the rainy seasons, low water velocity, and weak wave action, whereas *H. major* is found in the offshore islands (Nguyen et al., 2021b). “*Halophila major* SL type” collected from Nha Trang Bay showed morphological characters consistent with *H. major*; however, Nguyen et al. (2021a) suggested that this population may be the cross-hybridization between *H. ovalis* and *H. major*. Based on phylogenetic analysis of ITS1-5.8S-ITS2, Nguyen et al. (2021a) depicted that *H. major*, *H. ovalis*, and putative hybridization (*H. major* SL type) grouped into three districted clades (**Figure 3**). The cross-hybridization between *H. ovalis* and *H. major* was reported from materials collected at Sri Lankan waters (Liu et al., 2020) and may be “*Halophila ovalis* Red Sea type” at the Red Sea (Nguyen et al., 2018). Hybridization has been documented in other seagrass species, such as *Posidonia* (Sinclair et al., 2019) and *Halodule* (Ito and Tanaka, 2011). Hence, the genetic relationship between *H. ovalis*, *H. major*, *H. ovalis* Red Sea type, *H. major* SL type, and the hybridization from Sri Lankan waters should be clarified.

## Genetic diversity and population structures of selected species of the Hydrocharitaceae family

The seagrass species members of the Hydrocharitaceae in Viet Nam represent a great opportunity to study genetic structure and genetic diversity. Below, we discuss some recent studies that investigated genetic components of seagrass meadows in Viet Nam. For instance, Nguyen and Papenbrock (2019) demonstrated a reduced genetic diversity and a genetic differentiation between the lagoon sites versus the open sea sites for *E. acoroides* beds occurring along the coast of the Khanh Hoa Province. Based on the results from studying eight populations of *E. acoroides* using 11 polymorphic microsatellite loci along the South-Central Coast of Viet Nam, Dierick et al. (2021) reported clonal richness and structure, genetic diversity, and levels of dispersal within and between eight populations of *E. acoroides* in four lagoons along the South Central Coast of Viet Nam. The authors showed that lagoons were strongly differentiated and may act as barriers to gene flow and that large resistant genets contribute to the resilience of *E. acoroides* meadows under high levels of disturbance.

**Figure 4:**
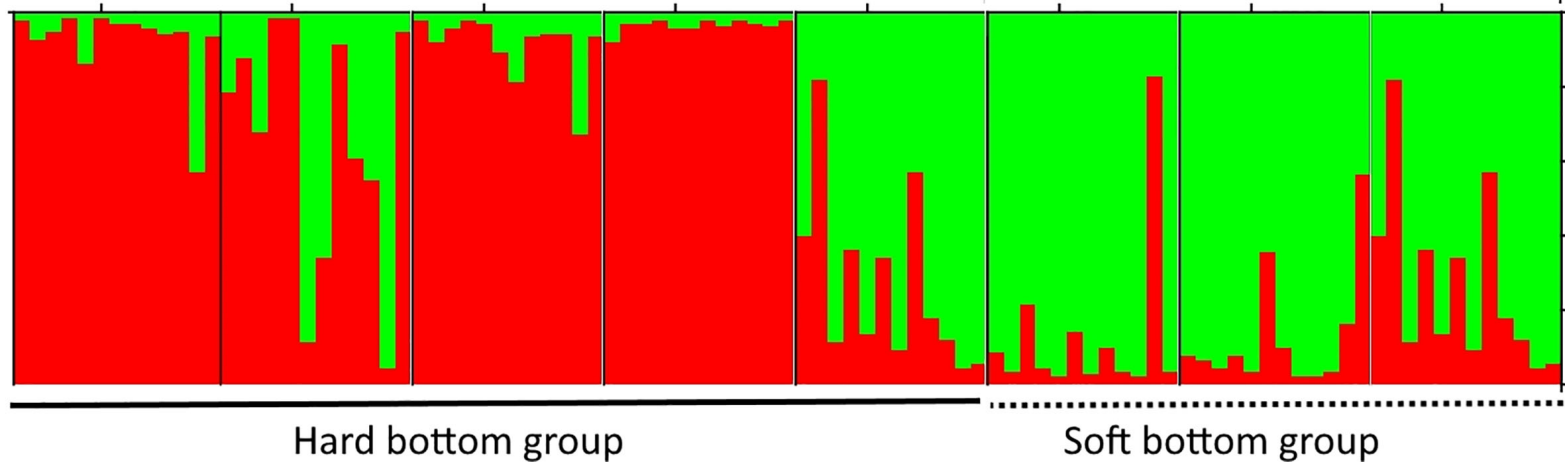
The cluster analysis of the *Thalassia hemprichii* population from southern Viet Nam based on nine microsatellite loci. Eight populations were divided into two groups consisting of the open sea and lagoon clusters. Adapted from Nguyen et al. (2022).

*Thalassia hemprichii* (Ehrenberg) Ascherson is another member of Hydrocharitaceae, which is quite common and widely distributed in the Tropical Indo-Pacific, including the east coast of Africa and the Red Sea (Ferrer-Gallego and Boisset, 2015). This species was used for several studies on genetic diversity, population structure, and gene flow among the populations in the Pacific and Indian Oceans (Hernawan et al., 2017; Wainwright et al., 2018; Jahnke et al., 2019b). However, reports on the genetic diversity and population structure of *T. hemprichii* in Viet Nam is very limited with only one study done so far (Nguyen et al., 2022). By using 10 loci of microsatellite markers, Nguyen et al. (2022) showed that eight populations were separated into two groups in agreement with the two different habitat types (hard and soft bottoms) (**Figure 4**), and the western boundary currents in the South China Sea influence the gene flow among *T. hemprichii* populations in southern Viet Nam. The authors also reveal that four populations with a high relative genetic contribution value should have the priority to be conserved (Nguyen et al., 2022).

**Figure 5:**
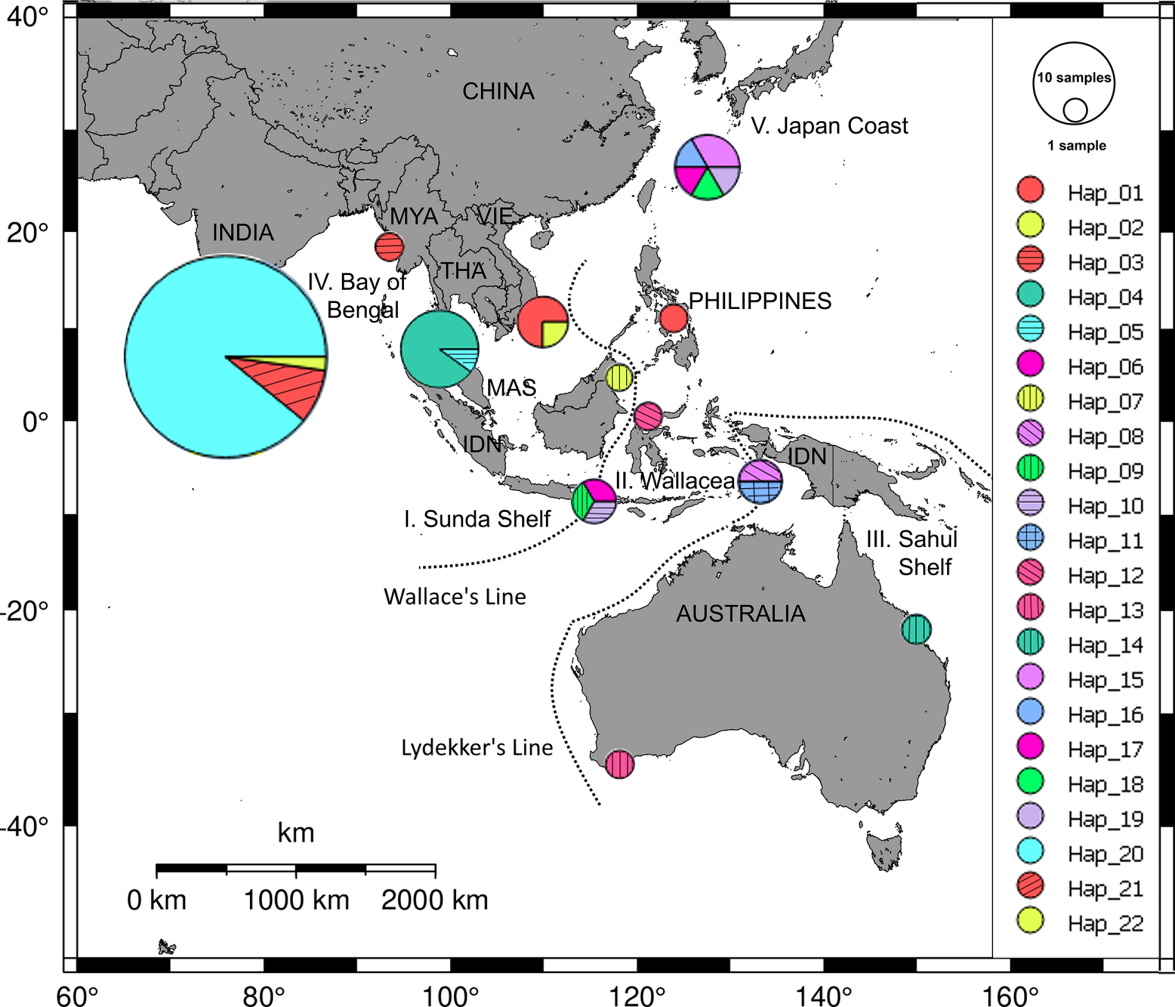
Distribution of haplotype frequency of *Halophila major*. Twenty-two haplotypes were found from dataset of 69 ITS sequences of *H. major* collected in five geographic regions: Sunda Shelf (I), Wallacea (II), Sahul Shelf (III), Bay of Bengal (IV), and coast of Japan (V). Source: Nguyen et al. (2021b).

For the members of the genus *Halophila*, *H. ovalis* is widely distributed in the Indo-Pacific region (Liu and Hsu, 2021). By using five loci of microsatellite markers, Nguyen et al. (2014) indicated that the genetic distances between southern Viet Nam and Gulf of Thailand were lower than other regions (Malaysia, Hong Kong, Andaman Sea). For the haplotype diversity of *H. ovalis*, there are seven haplotypes in the SEA (Nguyen et al., 2018). Along the coast of Viet Nam, the internal transcribed spacer (ITS) analysis indicated that there is only one haplotype, although sample collections were carried in five different locations (Nguyen et al., 2018). It is lower than in other neighbor countries in SEA such as Malaysia and Indonesia. *Halophila major*, the sister species of *H. ovalis*, was reported in offshore islands in Viet Nam. By using rDNA marker (ITS1-5.8S-ITS2), Nguyen et al. (2021b) depicted the haplotype network of *H. major* in the world (**Figure 5**). There are two haplotypes in southern Viet Nam, one of them is common to those from the Philippines. This number is much lower than in the Wallacea region where eight haplotypes were found (Nguyen et al., 2021b). Another member of the genus *Halophila*, *H. beccarii*, on the other hand, has been listed as a vulnerable species on the IUCN Red List of threatened seagrass species (Short et al., 2011), and it has been locally extirpated in the Philippines. Phan et al. (2017) revealed the low level of genetic and clonal diversity in *H. beccarii* in a Viet Nam lagoon habitat, and sexual reproduction is an important mode besides asexual regrowth in maintaining *H. beccarii* meadows.

## Seagrass conservation and management

The Fisheries Law of Viet Nam issued in 2017 included provisions for the protection and growth of aquatic resources, including marine conservation, in the sense of sustainable fisheries development and international integration (Government of Viet Nam, 2017). So far, the approval of the strategy for management of systems of special-use forests, marine reserves, and internal water reserves of Viet Nam through 2020, with a vision toward 2030 was issued in 2014 (Government of Viet Nam, 2014). In Viet Nam, 12 marine protected areas (MPAs) from 10 provinces/cities have been created and operationalized since 2005. It indicated that 35 main seagrass beds are out of protection. Unfortunately, huge seagrass beds including Tam Giang-Cau Hai lagoon in region 2, and seagrass beds along the coast of Khanh Hoa (region 3) are still under threat. As an International Union for Conservation of Nature (IUCN) category-II park, established in 2007, the Phu Quoc MPA (region 4) protects seagrass and coral-reef-based ecosystems (Kien Giang PCC, 2007). However, the results of Tran et al. (2022) showed that the protection of fishes provided by Phu Quoc MPA was ineffective due to lack of difference in species, and functional composition of fish communities was similar between protected and unprotected areas. Therefore, we need further action to optimize MPA design and management to meet conservation goals of seagrass meadows. More potential marine protection areas that include important seagrass beds should be added to the Vietnamese MPA system.

Currently, the management models of seagrass ecosystems in Viet Nam are mostly integrated into the integrated coastal management models to solve the problems of weaknesses that exist in the management, exploitation, and use of natural resources and environmental protection in coastal areas. The model of integrated coastal zone management, by Tran (2011) , is divided according to space separating the western coastal area of Tonkin Gulf; the northern coastal area (Quang Ninh–Ninh Binh), the northeastern coastal sub-region (Quang Ninh–Hai Phong), and the coastal area of Hai Phong (the city directly under the central government, the development center of the northern coastal region). This study combined three models of integrated management of the western coastal area of Tonkin Gulf, including managing, rational using of natural resources, and conserving nature and biodiversity; managing, preventing pollution, natural disasters, and environmental incidents; and strengthening institutions, policies, and raising awareness and responsibility for protecting natural resources—environment. In particular, the issues of natural, cultural, and biodiversity conservation include (i) biodiversity conservation, with focus on species diversity and rare and endangered species; (ii) protection and restoration of habitats and ecosystems, with focus on mangroves, coral reefs, seagrass beds, and sandy beaches; (iii) protection of natural landscapes including sea—islands, peninsulas, estuaries, and coastal zones; (iv) planning, construction, and management of nature reserves, including natural heritage sites, biosphere reserves, wetlands of national and international importance, marine protected areas, natural wonders, and natural landscapes (Khanh Ni et al., 2020; Tran, 2011).

Several models of seagrass ecosystem management were integrated into the integrated coastal zone management program in other areas, including the integrated management model of the coastal area in the North Central and Central Coast regions until 2010 and oriented to 2020 according to the Decision No. 158/2007/QĐ-TTg to strengthen management, protection, and utilization capacity of natural resources and environment protection for sustainable development of provinces and cities, and model of community-based and co-management of habitat marine resources and coastal ecosystems, mainly in the form of pilot-model projects implemented by domestic and international organizations (Government of Viet Nam, 2007). Presently, many coastal provinces have implemented the co-management and eco/community tourism models in the marine protected areas (e.g., Quang Ninh, Nam Dinh, and Quang Nam provinces) and co-management of exploiting and protecting aquatic resources (e.g., Quang Ninh, Nam Dinh, Thanh Hoa, Ha Tinh, Thua Thien Hue, Quang Ngai, and Binh Thuan provinces). Therefore, these successful models have been documented and expanded, such as the co-management model in Cu Lao Cham marine protected area, which was implemented from 2011 to the present. This management model has effectively managed resources based on the coordination between local community and management authorities, in which the authorities share management responsibilities with stakeholders and thereby contributing to the protection of coral and seagrass ecosystems (Hoang et al, 2020; Tran, 2011). On the other hand, the local authorities have oriented toward integrated management of seas and islands. However, their roles and responsibilities on the current planning and management practices are still undefined. There is no specific management model for each ecosystem, especially seagrass ecosystems.

Recently, there are very few distinct models of seagrass ecosystem management. One of the projects have been implemented by the Centre for Marinelife Conservation and Community Development (MCD) titled “Strengthening marine ecosystems management and developing local community livelihood, responding to climate change” in four main areas: Giao Thuy-Nam Dinh commune, Nam Phu-Thai Binh commune, Phu Long-Hai Phong commune, and Van Hung-Khanh Hoa commune. MCD builds a combination of community-based livelihood and resource management models such as the model of co-management of protected areas in order to utilize marine resources and sustainably develop those resources according to local regulations and management plans and the community model of sustainable fisheries to support the livelihoods of coastal communities in an environment-friendly practices and without destroying marine ecosystems and marine resources. MCD also diversifies livelihoods, increases income for coastal community, and reduces exploitation pressure on marine resources by developing local ecotourism models (MCD, 2013).

The application of the seagrass ecosystem management models can contribute to the management of exploitation and utilization on coastal resources in the coastal provinces, leading to gradual enhancement of the capacity of climate change adaptation in the future. However, these conservation models are few and mainly implemented in the form of pilot projects by domestic and international organizations.

## Future work

Mapping of seagrass beds in the mainland and estimation of the total cover have been done. However, the information on seagrass beds from the offshore islands, for example, some archipelagos in the Gulf of Thailand, is still lacking. Therefore, analyzing and mapping seagrass beds from a few offshore islands in southern Viet Nam are needed. Available remote sensing data are very efficient in the determination of sea grass beds coverage. However, for the species determination within seagrass beds, more in-depth methods need to be applied. The long-term monitoring of seagrass beds will be carried out to extend our understanding of the development and decline of seagrass ecosystems. For the species diversity, the putative hybridization between *H. ovalis* and *H. major* found from Nha Trang Bay should be clarified by using more plastid and nuclear DNA, leaf morphological dimension, and microsatellite DNA loci. Seagrasses are widely used to monitor heavy metal pollution in the nearshore environment and to bio-monitor metal and non-metal contamination in the marine ecosystem. However, only *E. acoroides* was studied in detail from Viet Nam. Therefore, more species should be included. The evaluation of seagrass ecosystem services is also an important step to demonstrate their usefulness to the ecosystem and to humans. Seagrasses can sequester significant amounts of carbon and store it as organic carbon in the sediments for a long time. There is still a need to evaluate the total C*_org_* stock from extensive seagrass beds. Sufficient genetic diversity seems to be a key factor to evaluate the health of the seagrass population. In Viet Nam, most of the studies focus onto some members of Hydrocharitaceae. Hence, more species of Cymodoceaceae and Zosteraceae should be carried out. More molecular studies on seagrass response to environmental changes, epigenetics, and holobionts are critical in our planning in the future. Finally, it is important to call for action, especially to speak with stakeholders and policy makers for better management of seagrass beds in Viet Nam.

This review clarified the recent distribution of seagrass from Viet Nam with 156.1 km^2^. Change detection of seagrass beds was presented from three specific areas. There are 15 species including putative hybridization of *Halophila*. Only one haplotype of *H. ovalis* and two haplotypes of *H. major* were found along the coast of Viet Nam. Genetic diversity and population structure from four species within Hydrocharitaceae were presented. In addition, other aspects of blue carbon storage and phytoremediation processes of seagrass from Viet Nam were also reported.

## Author contributions

X-VN and JP contributed to conceptualization, writing the original draft, review, and editing. TP, N-TN, X-TN, and T-HN contributed to genetic diversity and taxonomy. V-KL and V-LC contributed to mapping and change detection of seagrass. C-TH contributed to seagrass management. TP and V-LC contributed to blue carbon. X-VN contributed to biodiversity within seagrass. HMN and TP contributed to ecology and physiology. M-NN-T and V-HD contributed to project administration. JP, HMN, and MT contributed to review and editing. All authors contributed to the article and approved the submitted version.

## Funding

This work was supported by Viet Nam Academy of Science and Technology, grant number TĐĐTB0.04/21-23.

## Acknowledgments

We thank project 106.06-2020.40 of the National Foundation for Science and Technology Development (NAFOSTED), project VAST06.01/22-23 of Viet Nam Academy of Science and Technology for sharing information. We also thank to JSPS Core-to-Core Program CREPSUM JPJSCCB20200009. This paper is a contribution to celebrate the 100th anniversary of the Institute of Oceanography, Nha Trang Viet Nam Academy of Science and Technology.

## Conflict of interest

The authors declare that the research was conducted in the absence of any commercial or financial relationships that could be construed as a potential conflict of interest.

## Publisher’s note

All claims expressed in this article are solely those of the authors and do not necessarily represent those of their affiliated organizations, or those of the publisher, the editors and the reviewers. Any product that may be evaluated in this article, or claim that may be made by its manufacturer, is not guaranteed or endorsed by the publisher.
